# Effect of remission, clinical remission with active serology, and glucocorticoid dosage on the pregnancy outcome of pregnant patients with systemic lupus erythematosus

**DOI:** 10.1186/s13075-024-03298-6

**Published:** 2024-03-09

**Authors:** Takehiro Nakai, Nanase Honda, Eri Soga, Sho Fukui, Ayako Kitada, Naoto Yokogawa, Masato Okada

**Affiliations:** 1 Immuno-Rheumatology Center, St. Luke’s International Hospital, 9-1 Akashi-cho, Chuo-ku, Tokyo, Japan; 2 Department of Rheumatic Diseases, Tokyo Metropolitan Tama Medical Center, Tokyo, Japan; 3 Department of Obstetrics and Gynecology, Tokyo Metropolitan Tama Medical Center, Tokyo, Japan; 4 Department of General Medicine, Kyorin University School of Medicine, Tokyo, Japan; 5 Division of Rheumatology, Inflammation, and Immunity, Department of Medicine, Brigham and Women’s Hospital and Harvard Medical School, Boston, MA USA; 6 Department of Rheumatology, Institute of Medicine, University of Tsukuba, Ibaraki, Japan

**Keywords:** Systemic lupus erythematosus, Pregnancy, Remission, Clinical remission with serological activity, Glucocorticoid

## Abstract

**Background:**

Remission is a key treatment target in systemic lupus erythematosus (SLE) management. Given the direct correlation between lupus flares and elevated risks of adverse pregnancy outcomes (APOs), securing remission before conception becomes crucial. However, the association between clinical remission with active serology, and the risk of APOs is not thoroughly understood. Additionally, determining the optimal glucocorticoid dosage during pregnancy to mitigate APO risks remains under-researched. This study investigated the risk of APOs in relation to remission/serological activity status in patients in clinical remission/glucocorticoid dosage.

**Methods:**

Pregnant patients with SLE, who were followed up at two Japanese tertiary referral centers, and had their remission status assessed at conception, were included in this study. We categorized the patients into two groups based on whether they achieved Zen/Doria remission at conception and analyzed the APO ratio. We also examined the influence of serological activity in pregnant patients with clinical remission and analyzed the optimal glucocorticoid dosage to minimize the APO ratio.

**Results:**

Of the 96 pregnancies included, 59 achieved remission at conception. Pregnant patients who achieved remission showed a significant decrease in the APO ratio compared with those who did not. (overall APO: odds ratio (OR) 0.27, 95% confidence interval (CI) 0.11–0.65, *p* < 0.01, maternal APO: OR 0.34, 95%CI 0.13–0.85, *p* = 0.021, neonatal APO: OR 0.39, 95%CI 0.17–0.90, *p* = 0.028). Conversely, no statistical difference was observed in the APO ratio based on serological activity in pregnant patients with clinical remission. (overall APO: OR 0.62, 95%CI 0.21–1.79, *p* = 0.37, maternal APO: OR 1.25, 95%CI 0.32–4.85, *p* = 0.75, neonatal APO: OR 0.83, 95%CI 0.29–2.39, *p* = 0.73). A glucocorticoid dose of prednisolone equivalent ≥ 7.5 mg/day at conception correlated with increased APO. (overall APO: OR 3.01, 95%CI 1.23–7.39, *p* = 0.016, neonatal APO: OR 2.98, 95% CI:1.23–7.22, *p* = 0.016).

**Conclusions:**

Even with active serology, achieving clinical remission can be a clinical target for reducing APOs in patients who wish to conceive. In addition, if clinically feasible, reducing the glucocorticoid dosage to < 7.5 mg/day before conception could be another predictive factor.

**Supplementary Information:**

The online version contains supplementary material available at 10.1186/s13075-024-03298-6.

## Background

Remission is a primary objective in systemic lupus erythematosus (SLE) treatment as it is associated with reduced organ damage and improved mortality rates [1–3]. Regarding pregnancy care, active disease increases adverse pregnancy outcomes (APOs), including lupus flares during pregnancy. Achieving low disease activity state and remission is associated with an improved APO ratio [4, 5].

LLDAS (lupus low disease activity state) and DORIS (Definitions of Remission in SLE) remission are commonly used to assess the relationship between disease remission and APO; however, data on their relationship with Zen/Doria remission remain limited; therefore, this study investigated this association.

Moreover, in SLE management, maintaining a serologically active yet clinically quiescent state is associated with reduced organ damage and flare rates compared with individuals without remission [3, 6]. By achieving both clinical and serological remission, future flare-ups and mortality rates can be further reduced [1, 6, 7],

As lupus flares are associated with increased APOs [8], achieving both serological and clinical remission before pregnancy is desirable. However, it can be challenging to attain this state due to the limited time available for conception and the restricted choice of medication compatible with use during pregnancy. Sometimes, it is necessary to consider pregnancy in patients with clinical remission but with serologically active cases. However, there is currently no available data regarding pregnancy outcomes in such cases.

Furthermore, it is recommended that glucocorticoid dosage should be minimized in the management of SLE, ideally to less than 5 mg/day and if possible discontinue [9–12], as glucocorticoids are associated with organ damage [13–15]. Reducing glucocorticoid dose is also recommended in pregnancy care to improve pregnancy outcomes and reduce associated complications. Nonetheless, in the context of managing pregnancy concomitant with SLE, certain medications are contraindicated, rendering glucocorticoids a principal therapeutic option during gestation. Despite these considerations, there is still much to be investigated, particularly concerning the optimal cut-off glucocorticoid dosage to reduce the APO ratio while reducing the risk of SLE flare during pregnancy.

Therefore, we examined the risk of APOs in the following variables: the status of remission, the serological activity in individuals experiencing clinical remission, and the glucocorticoid dosage.

## Methods

### Study design

We conducted a retrospective analysis using the complete health records of patients with SLE who received treatment at Tokyo Metropolitan Tama Medical Center (Tokyo, Japan) and St. Luke’s International Hospital (Tokyo, Japan) between April 2010 and September 2022. Patients with complete follow-up data during pregnancy and assessment of remission at conception were included. We excluded patients who lack consistent maternity care throughout gestation in our centers, whose data on pregnancy outcomes were lacking, or those who declined participation in the study.

The patients were stratified based on the attainment of remission at conception, and the APO ratios were compared. The study was approved by the Ethics Committee of St. Luke’s International Hospital, and written informed consent was obtained from all participants (approval No. 22-R141).

### SLE diagnosis

The diagnosis of SLE was based on three major classification criteria: 1997 American College of Rheumatology (ACR) criteria, Systemic Lupus International Collaborating Clinics 2012 criteria, and 2019 European League Against Rheumatism (EULAR)/ACR criteria. We considered patients as having SLE if they satisfied any one of these three sets of guidelines as over-reliance on single classification criteria may occasionally overlook genuine SLE cases [16–19].

### Data collection

We collected data on demographics, the duration between SLE onset and conception, organ manifestations, immunological profiles, and treatment regimens during pregnancy. We also gathered data on maternal and neonatal pregnancy outcomes.

### Definition of SLE flare

The aggravation of SLE symptoms observed over the preceding four weeks, relative to earlier evaluations and characterized by a British Isles Lupus Assessment Group (BILAG) Category A in at least one organ system, was deemed an SLE flare.

### Definition of APOs

We collected data on four types of APOs: overall, maternal, neonatal, and PROMISSE (Predictors of Pregnancy Outcome: Biomarkers in Antiphospholipid Antibody Syndrome and SLE) APO. Maternal APO was defined as the occurrence of at least one of the following: SLE flares during pregnancy, gestational diabetes mellitus, hypertensive disorder of pregnancy, preeclampsia, HELLP syndrome, or maternal death during pregnancy. Neonatal APO was defined as neonates with at least one of the following: preterm birth (live birth before 37 weeks of gestation), spontaneous abortion (fetal death at < 22 weeks of gestation), stillbirth (fetal death at ≥ 22 weeks of gestation), low birth weight (birth weight < 2500 g), small for gestational age (SGA) (body weight and/or height below the 10th percentile for gestational age), Apgar score < 7 at 1 or 5 min, and major malformations. Overall APO was defined as any maternal and/or neonatal APO. PROMISSE APO was defined as fetal death after 12 weeks of gestation, neonatal death before hospital discharge, preterm delivery or termination of pregnancy before 36 weeks, or SGA [20].

### Definition of SLE remission

Zen/Doria remission was employed as an indicator of disease remission [21]. “Complete remission” was defined as the absence of clinical and serological activity without prednisolone (PSL)/immunosuppressants. “Clinical remission off corticosteroids” was defined as the absence of clinical activity without glucocorticoids, although the use of immunosuppressants was permissible in this group. “Clinical remission on corticosteroids” was defined as the absence of clinical activity using low-dose glucocorticoids with or without the adjunct of immunosuppressants.

Furthermore, pregnant individuals in remission were divided into two groups based on the attainment of serological negativity: those with clinical remission but active serology and those with both clinical and serological remission.

### Statistical analysis

Categorical data were presented as numbers and percentages, whereas continuous data were expressed as median values and interquartile ranges. Fisher’s exact and Mann–Whitney U tests were employed to compare qualitative and continuous variables, respectively. Univariate logistic regression models were used to calculate the odds ratios (OR) for each APO based on the achievement of each remission type at conception and to evaluate the impact of serological remission in clinically remitted patients. Multivariate logistic regression analysis was performed to analyze primary pregnancy outcomes, namely overall/maternal/neonatal/PROMISSE APOs, using previously identified variables linked with an increased APO ratio. Specifically, these variables encompass the presence of renal manifestation, employment of hydroxychloroquine at conception, and use of aspirin at conception [22–25]. In addition, Receiver Operating Characteristic (ROC) curve analysis was conducted to determine the optimal cut-off glucocorticoid dose for each APO. The area under the curve (AUC) was calculated, with AUC values ranging from 0.5 to 0.7 indicating low diagnostic accuracy, AUC values from 0.7 to 0.9 indicating moderate diagnostic accuracy, and AUC > 0.9 indicating high diagnostic accuracy [26].

All statistical analyses were performed using EZR (version 2.7–1; Saitama Medical Center, Jichi Medical University, Saitama, Japan), a graphical user interface for R (The R Foundation for Statistical Computing, Vienna, Austria). Statistical significance was set at *p* < 0.05.

## Results

### Population characteristics

This study initially encompassed 124 pregnancies in 97 women under observation in two institutions. Of these, 28 pregnancies in 21 women lacked uniform maternity care during gestation within our centers. Aligning with our primary aim of elucidating the APO ratio in patients receiving comprehensive maternity care throughout their gestation, these cases were subsequently excluded from the analysis.

Finally, we included 96 pregnancies in 76 pregnant women with assessment on remission achievement at conception.

Of them, 41 achieved clinical remission on corticosteroids, 7 achieved clinical remission off corticosteroids, and 11 achieved complete remission. Thirty-seven pregnancies did not achieve remission at conception (Supplementary Figure S1). No statistically significant differences were observed in age at conception, body mass index (BMI), disease duration, rate of hypertensive medication use before pregnancy, multiparity, or rate of infertility treatment based on remission achievement.

Within the remission cohort, 98.3% of the pregnancy was planned, whereas in the non-remission cohort, 73.0% of the pregnancy was planned. Notably, there was a single case of unplanned pregnancy within the remission cohort, occurring in a patient who conceived while undergoing treatment with mizoribine.

The prevalence of renal manifestations tended to be lower in patients with remission than in those without remission (remission vs. no remission: 20.3% vs. 35.1%, *p* = 0.15). The two groups had no statistically significant differences in the prevalence of other organ manifestations.

There were no significant differences between the two groups regarding immunological profiles, except for the prevalence of anti-dsDNA antibodies (74.6% vs. 51.4%, *p* = 0.027) (Table 1 and Supplementary Table S1).

**Table 1:** Baseline characteristics based on the achievement of remission at conception

Factor	no remission	remission	*p*-value
*n*	37	59	
***Epidemiological findings***			
Age at conception (y.o)	34.0 [31.0, 36.0]	33.0 [29.0, 35.5]	0.13
Japanese ethnicity (%)	33 (89.2)	53 (89.8)	1.0
BMI	19.7 [19.0, 21.3]	20.0 [18.0, 21.2]	0.41
Duration of SLE (days)	2943.0 [1893.0, 5198.0]	2416.0 [1387.5, 4226.5]	0.31
Smoking history (%)	3 (8.1)	4 (6.8)	1.0
Previous spontaneous abortion (%)	6 (16.2)	12 (20.3)	0.79
Previous anti-hypertensive med use (%)	4 (10.8)	2 (3.4)	0.20
Multiparous (%)	15 (40.5)	21 (36.2)	0.67
Infertility treatment (%)	10 (27.0)	17 (28.8)	1.0
**Planned pregnancy (%)**	**27 (73.0)**	**58 (98.3)**	**< 0.01**
Thrombocytopenia at conception (%)	2 (5.4)	0 (0.0)	0.14
**Any flare at conception (%)**	**6 (16.2)**	**0 (0.0)**	**< 0.01**
**Zen/Doria remission at conception (%)**	**0 (0.0)**	**59 (100.0)**	**< 0.01**
***Organ manifestation***			
Joint/muscular manifestation (%)	26 (70.3)	38 (64.4)	0.66
Skin/mucocutaneous manifestation (%)	27 (73.0)	44 (74.6)	1.0
Renal manifestation (%)	13 (35.1)	12 (20.3)	0.15
Lupus nephritis class III/IV (%)	5 (13.5)	4 (6.8)	0.30
Serositis (%)	7 (18.9)	15 (25.4)	0.62
Neurological manifestation (%)	2 (5.4)	7 (11.9)	0.48
Hematological manifestation (%)	28 (75.7)	53 (89.8)	0.084
***Immunological profile***			
**Anti-DNA Ab (%)**	**19 (51.4)**	**44 (74.6)**	**0.027**
Anti-RNP Ab (%)	12 (48.0)	18 (36.7)	0.45
Anti-Sm Ab (%)	8 (25.8)	23 (40.4)	0.24
Anti-SSA Ab (%)	21 (56.8)	40 (69.0)	0.28
Anti-SSB Ab (%)	2 (7.7)	10 (21.3)	0.19
LAC (%)	4 (11.1)	8 (14.0)	0.76
Anti-CL Ab (%)	5 (15.2)	17 (29.3)	0.20
Anti-CLβ2GPI Ab (%)	3 (8.3)	5 (8.6)	1.0
Low C3 (%)	23 (65.7)	40 (67.8)	1.0
Low C4 (%)	30 (85.7)	47 (79.7)	0.58

Ab, antibody; BMI, body mass index; CL, cardiolipin; LAC, lupus anticoagulant; SLE, systemic lupus erythematosus

### Treatment regimen at conception

The glucocorticoid dosage was lower in individuals with remission than in those without remission (PSL equivalent: 4.00 [0.00, 5.00] mg/day vs. 10.00 [8.00, 11.00] mg/day, *p* < 0.01). A higher proportion of pregnant patients in remission used hydroxychloroquine and aspirin, and a lower proportion used tacrolimus compared to those not in remission (hydroxychloroquine usage: 54.2% vs. 37.8%, *p* = 0.14; aspirin usage: 50.8% vs. 32.4%, *p* = 0.093; tacrolimus usage: 22.0% vs. 35.1%, *p* = 0.24) (Table 2 and Supplementary Tables S2, S3). Notably, one pregnant patient in remission was administered mizoribine, and one patient without remission was administered mycophenolate mofetil at conception. Both of the pregnancies were unplanned, and iatrogenic abortion was performed after careful discussions with the attending doctor. In addition, one pregnant patient in remission and two without remission used belimumab at conception. However, all of them discontinued belimumab after conception due to the lack of sufficient safety data on belimumab during pregnancy [27–33], and various studies have demonstrated the possibility of belimumab discontinuation [34, 35].

**Table 2:** Treatment regimen at conception

	Zen/Doria remission		
	no remission	remission	*p*-value
*n*	37	59	
**GC (mg/day)**	**10.00 [8.00, 11.00]**	**4.00 [0.00, 5.00]**	**< 0.01**
HCQ (%)	14 (37.8)	32 (54.2)	0.14
Tac (%)	13 (35.1)	13 (22.0)	0.24
CyA (%)	0 (0.0)	2 (3.4)	0.52
AZA (%)	2 (5.4)	4 (6.8)	1.0
MMF (%)	1 (2.7)	0 (0.0)	0.39
MZR (%)	0 (0.0)	1 (1.7)	1.0
MTX (%)	0 (0.0)	0 (0.0)	NA
BEL (%)	2 (5.4)	1 (1.7)	0.56
RTX/CY/PE/IVIg (%)	0 (0.0)	0 (0.0)	NA
aspirin (%)	12 (32.4)	30 (50.8)	0.093

AZA, azathioprine; BEL, belimumab; CY, cyclophosphamide; CyA, cyclosporine; GC, glucocorticoid; HCQ, hydroxychloroquine; IVIg, intravenous immunoglobulin; MMF, mycophenolate mofetil; MTX, methotrexate; MZR, MZR; NA, not available; PE, plasma exchange; PSL, prednisolone; RTX, rituximab; Tac, tacrolimus

### Zen/Doria remission and APO ratio

Pregnant women with remission demonstrated a lower frequency of overall, maternal, and neonatal APOs, compared with those without remission (overall APO: 39.0% vs. 70.3%, *p* < 0.01; maternal APO: 18.6% vs. 40.5%, *p* = 0.032; neonatal APO: 39.0% vs. 62.2%, *p* = 0.036). In addition, those with remission had a lower flare rate during pregnancy, a longer total duration of gestation, and higher birth weight than did those without remission (flare during pregnancy: 3.4% vs. 21.6%; total duration of gestation: 268.0 [262.0, 276.0] days vs. 262.0 [242.0, 271.0] days; weight at birth: 2716.0 [2476.5, 3013.8] g vs. 2472.0 [2202.0, 2896.0] g). Pregnant women with remission also showed a lower incidence of neonates with low birth weight than did those without remission (low birth weight: 27.8% vs. 51.7%). There was a trend towards a higher live birth rate in those with remission than those without remission (91.5% vs. 78.4%).

The logistic regression model also indicated that achieving remission was associated with a reduced prevalence of overall, maternal, and neonatal APOs compared with those without remission (overall APO: OR 0.27, 95% clinical interval (CI) 0.11–0.65, *p* < 0.01; maternal APO: OR 0.34, 95%CI 0.13–0.85, *p* = 0.021; neonatal APO: OR 0.39, 95%CI 0.17–0.90, *p* = 0.028) (Table 3).

**Table 3:** Adverse pregnancy outcome ratio according to the achievement of remission at conception

Factor	Zen/Doria remission			Logistic regression model (univariate analysis)			Logistic regression model (multivariate analysis)		
	no remission	remission	*p*-value	OR^a^	95%CI	*P* value	aOR^b^	95% CI	*P* value
*n*	37	59							
**Overall APO (%)**	**26 (70.3)**	**23 (39.0)**	**< 0.01**	**0.27**	**0.11–0.65**	**< 0.01**	**0.28**	**0.11–0.70**	**< 0.01**
**Maternal APO (%)**	**15 (40.5)**	**11 (18.6)**	**0.032**	**0.34**	**0.13–0.85**	**0.021**	**0.33**	**0.12–090**	**0.030**
**Neonatal APO (%)**	**23 (62.2)**	**23 (39.0)**	**0.036**	**0.39**	**0.17–0.90**	**0.028**	**0.37**	**0.15–0.90**	**0.029**
PROMISSE APO (%)	10 (27.0)	12 (20.3)	0.47	0.69	0.26–1.81	0.45	0.64	0.23–1.76	0.38
Flare during pregnancy (%)	8 (21.6)	2 (3.4)		0.13	0.03–0.64				
Flare after delivery (%)	2 (6.7)	1 (1.9)		0.26	0.23–3.04				
Gestational DM (%)	6 (16.2)	4 (6.8)		0.38	0.10–1.43				
Preeclampsia (%)	3 (8.1)	3 (5.1)		0.61	0.12–3.18				
Hypertensive disorders in pregnancy (%)	7 (18.9)	6 (10.2)		0.49	0.15–1.58				
HELLP syndrome (%)	1 (2.7)	1 (1.7)		0.62	0.04–10.2				
Oligohydramnios (%)	6 (16.2)	2 (3.4)		0.19	0.04–0.97				
Maternal death (%)	0 (0.0)	0 (0.0)		NA	NA				
Live birth (%)※	29 (78.4)	54 (91.5)		2.98	0.89–9.94				
Total duration of gestation (days)	262.0 [242.0, 271.0]	268.0 [262.0, 276.0]		NA	NA				
Preterm birth (%)	6 (18.2)	8 (14.8)		0.78	0.25–2.5				
Spontaneous abortion (%)	1 (2.8)	2 (3.4)		1.25	0.11–14.3				
Missed abortion (%)	3 (8.1)	1 (1.8)		0.20	0.02–2.02				
Iatrogenic abortion (%)	5 (13.5)	2 (3.4)		0.23	0.04–1.22				
Still birth (%)	0 (0.0)	0 (0.0)		NA	NA				
Height at birth (cm)	46.0 [43.8, 48.0]	48.0 [46.5, 49.5]		NA	NA				
Weight at birth (g)	2472.0 [2202.0, 2896.0]	2716.0 [2476.5, 3013.8]		NA	NA				
Low birth weight (%)	15 (51.7)	15 (27.8)		0.36	0.14–0.92				
SGA (%)	6 (20.7)	8 (14.8)		0.67	0.21–2.15				
Apgar score (1 m)	8.00 [8.00, 8.00]	8.00 [8.00, 8.00]		NA	NA				
Apgar Score (5 m)	9.00 [9.00, 9.00]	9.00 [9.00, 9.00]		NA	NA				
Apgar.score.1 m > 7 (%)	27 (93.1)	53 (98.1)		3.93	0.34–45.3				
Apgar.score.5 m > 7 (%)	29 (100.0)	54 (100.0)		NA	NA				
Major malformation	1 (3.4)	1 (1.9)		0.53	0.32–8.80				
Death of neonate	0 (0.0)	0 (0.0)		NA	NA				

a Odds ratio of Zen/Doria remission attainment for each APO

b adjusted odds ratio of Zen/Doria remission attainment for each APO

※ multivariate analysis adjusted for renal manifestation, hydroxychloroquine prescription, and aspirin prescription at conception

APO; adverse pregnancy outcome, DM; diabetes mellitus, NA; not applicable, OR; odds ratio, PROMISSE; Predictors of Pregnancy Outcome: Biomarkers in Antiphospholipid Antibody Syndrome and Systemic Lupus Erythematosus, SGA; small for gestational age

In addition, we performed multivariate logistic regression model analysis with variables reported as associated with the APO ratio, namely renal manifestation, hydroxychloroquine prescription at conception, and aspirin prescription at conception [22–25].

As shown in Table 3 and supplementary Table S4, after multivariate analysis, overall/maternal/neonatal APO showed a statistical decrease in patients with remission (overall APO: adjusted Odds ratio (aOR) 0.28, 95%CI 0.11–0.70, *p* < 0.01, maternal APO: aOR 0.33, 95%CI 0.12–0.90, *p* = 0.030, neonatal APO: aOR 0.37, 95%CI 0.15–0.90, *p* = 0.029) PROMISSE APO ratio showed decrease tendency in those with remission, but no statistical difference was noted (aOR 0.64, 95%CI 0.23–1.76, *p* = 0.38).

Furthermore, we subdivided the patients into three groups based on the remission achieved: clinical remission on corticosteroids, clinical remission off corticosteroids, and complete remission. The analysis revealed that all forms of remission were associated with a statistically significant decrease in the overall APO compared to those without remission (complete remission vs. clinical remission off corticosteroids vs. clinical remission on corticosteroids vs. no remission: overall APO: 36.4% vs. 28.6% vs. 41.5% vs. 70.3%, *p* = 0.025). In addition, those with any form of remission had a longer total duration of gestation and higher birth weight compared with those without remission (total duration of gestation: 270.0[253.0, 281.5] days vs. 276.0 [273.5, 278.5] days vs. 267.0 [262.0, 275.0] days vs. 262.0 [242.0, 271.0] days; weight at birth: 3004 [2574, 3176] g vs. 3120 [2727, 3230] g vs. 2658 [2452, 2925] g vs. 2472 [2202, 2896] g) (Supplementary Table S5). Furthermore, the logistic regression model demonstrated that any form of remission was associated with a decreased frequency of overall, maternal, and neonatal APOs (overall APO: complete remission vs. no remission: OR 0.24, 95%CI 0.06–1.00, *p* = 0.049; clinical remission off corticosteroids vs. no remission: OR = 0.17, 95%CI 0.03–1.01, *p* = 0.051; clinical remission on corticosteroids vs. no remission: OR 0.3, 95%CI 0.12–0.77, *p* = 0.012) (maternal APO: complete remission vs. no remission: OR 0.55, 95%CI 0.13–2.42, *p* = 0.43; clinical remission on corticosteroids vs. no remission: OR 0.36, 95%CI 0.13–0.98, *p* = 0.045; clinical remission off corticosteroids vs. no remission was not assessed because no maternal APO was noted in pregnant patients with clinical remission off corticosteroids) (neonatal APO: complete remission vs. no remission: OR 0.35, 95%CI 0.09–1.41, *p* = 0.14; clinical remission off corticosteroids vs. no remission: OR 0.24, 95%CI 0.04–1.43, *p* = 0.12; clinical remission on corticosteroids vs. no remission: OR 0.43, 95%CI 0.17–1.07, *p* = 0.070) (Supplementary Table S6).

### Clinical remission with active serology and APO

We examined the differences in the APO ratio between pregnant individuals in clinical remission with serological activity (positivity for anti-dsDNA antibody and/or hypocomplementemia) and those with both clinical and serological remission.

There were no statistically significant differences in age at conception, BMI, or organ manifestations. However, the duration of SLE was significantly shorter in patients with clinical and serological remission compared with those with clinical remission with active serology (clinical and serological remission vs. clinical remission with active serology: 1760 [1152, 3296] days vs. 2849 [2146, 4230] days, *p* = 0.047) (Supplementary Table S7).

Regarding the treatment regimen at conception, pregnant patients in clinical and serological remission had lower glucocorticoid dosages. The prescription rate of aspirin was higher in pregnant patients in clinical and serological remission compared with those with clinical remission and active serology (glucocorticoid dosage [PSL equivalent]: 0.50 [0.00, 5.00] mg/day vs. 4.50 [2.50, 5.00] mg/day, *p* = 0.018; aspirin usage: 58.3% vs. 45.7%, *p* = 0.43). The prescription rate of hydroxychloroquine was lower in individuals with clinical and serological remission than in those with clinical remission and active serology; however, no statistical difference was observed (45.8% vs. 60.0%, *p* = 0.30) (Supplementary Table S8). As shown in Table 4, no significant difference was observed in the APO ratio based on serological activity among pregnant patients in clinical remission (clinical and serological remission vs. clinical remission with active serology: overall APO: 45.8% vs. 34.3%, *p* = 0.42; maternal APO: 16.7% vs. 20.0%, *p* = 1.00; neonatal APO: 41.7% vs. 37.1%, *p* = 0.79; PROMISSE APO: 25.0% vs. 17.1%, *p* = 0.52; flare during pregnancy: 0.0% vs. 5.7%; flare after delivery: 0.0% vs. 3.1%).

**Table 4:** APO ratio in pregnant patients in clinical remission at conception based on the serological activity

Factor	pregnant in clinical remission at conception			Logistic regression model		
	clinical remission with active serology	clinical and serological remission	*p*-value	OR^a^	95% CI	*p*-value
*n*	35	24				
Overall APO (%)	12 (34.3)	11 (45.8)	0.42	0.62	0.21–1.79	0.37
Maternal APO (%)	7 (20.0)	4 (16.7)	1.00	1.25	0.32–4.85	0.75
Neonatal APO (%)	13 (37.1)	10 (41.7)	0.79	0.83	0.29–2.39	0.73
PROMISSE APO (%)	6 (17.1)	6 (25.0)	0.52	0.62	0.17–2.22	0.46
Flare during pregnancy (%)	2 (5.7)	0 (0.0)		NA	NA	
Flare after delivery (%)	1 (3.1)	0 (0.0)		NA	NA	
Gestational DM (%)	3 (8.6)	1 (4.2)		2.16	0.21–22.1	
Preeclampsia (%)	3 (8.6)	0 (0.0)		NA	NA	
Hypertensive disorders in pregnancy (%)	3 (8.6)	3 (12.5)		0.66	0.12–3.56	
HELLP syndrome (%)	1 (2.9)	0 (0.0)		NA	NA	
Oligohydramnios (%)	1 (2.9)	1 (4.3)		0.65	0.04–10.9	
Maternal death (%)	0 (0.0)	0 (0.0)		NA	NA	
Live birth (%)	32 (91.4)	22 (91.7)		0.97	0.15–6.30	
Total duration of gestation (days)	270.00 [263.50, 276.00]	267.50 [260.00, 273.75]		NA	NA	
Preterm birth (%)	4 (12.5)	4 (18.2)		0.64	0.14–2.90	
Spontaneous abortion (%)	2 (5.7)	0 (0.0)		NA	NA	
Missed abortion (%)	0 (0.0)	1 (4.3)		NA	NA	
Iatrogenic abortion (%)	1 (2.9)	1 (4.2)		0.68	0.04–11.4	
Still birth (%)	0 (0.0)	0 (0.0)		NA	NA	
Height at birth (cm)	48.00 [46.08, 49.10]	48.00 [47.00, 49.50]		NA	NA	
Weight at birth (g)	2716.00 [2539.50, 2935.00]	2776.00 [2419.00, 3062.00]		NA	NA	
Low birth weight (%)	7 (21.9)	8 (36.4)		0.49	0.16–1.64	
SGA (%)	4 (12.5)	4 (18.2)		0.64	0.14–2.9	
Apgar score (1 m)	8.00 [8.00, 8.00]	8.00 [7.25, 8.00]		NA	NA	
Apgar Score (5 m)	9.00 [9.00, 9.00]	9.00 [9.00, 9.00]		NA	NA	
Apgar.score.1 m > 7 (%)	31 (96.9)	22 (100.0)		NA	NA	
Apgar.score.5 m > 7 (%)	32 (100.0)	22 (100.0)		NA	NA	
Major malformation (%)	1 (3.1)	0 (0.0)		NA	NA	
Death of neonate (%)	0 (0.0)	0 (0.0)		NA	NA	

a Odds ratio of serological activity among patients with SLE who achieved clinical remission for each APO

APO; adverse pregnancy outcome, DM; diabetes mellitus, NA; not applicable, OR; odds ratio, PROMISSE; Predictors of Pregnancy Outcome: Biomarkers in Antiphospholipid Antibody Syndrome and Systemic Lupus Erythematosus, SGA; small for gestational age

Furthermore, the logistic regression model also revealed no significant differences in the frequency of any type of APOs (overall APO: OR 0.62, 95%CI: 0.21–1.79, *p* = 0.37; maternal APO: OR 1.25, 95%CI: 0.32–4.85, *p* = 0.75; neonatal APO: OR 0.83, 95%CI 0.29–2.39, *p* = 0.73; PROMISSE APO: OR 0.62, 95%CI 0.17–2.22, *p* = 0.46).

### Glucocorticoid dosage at conception and APOs

**Fig. 1:**
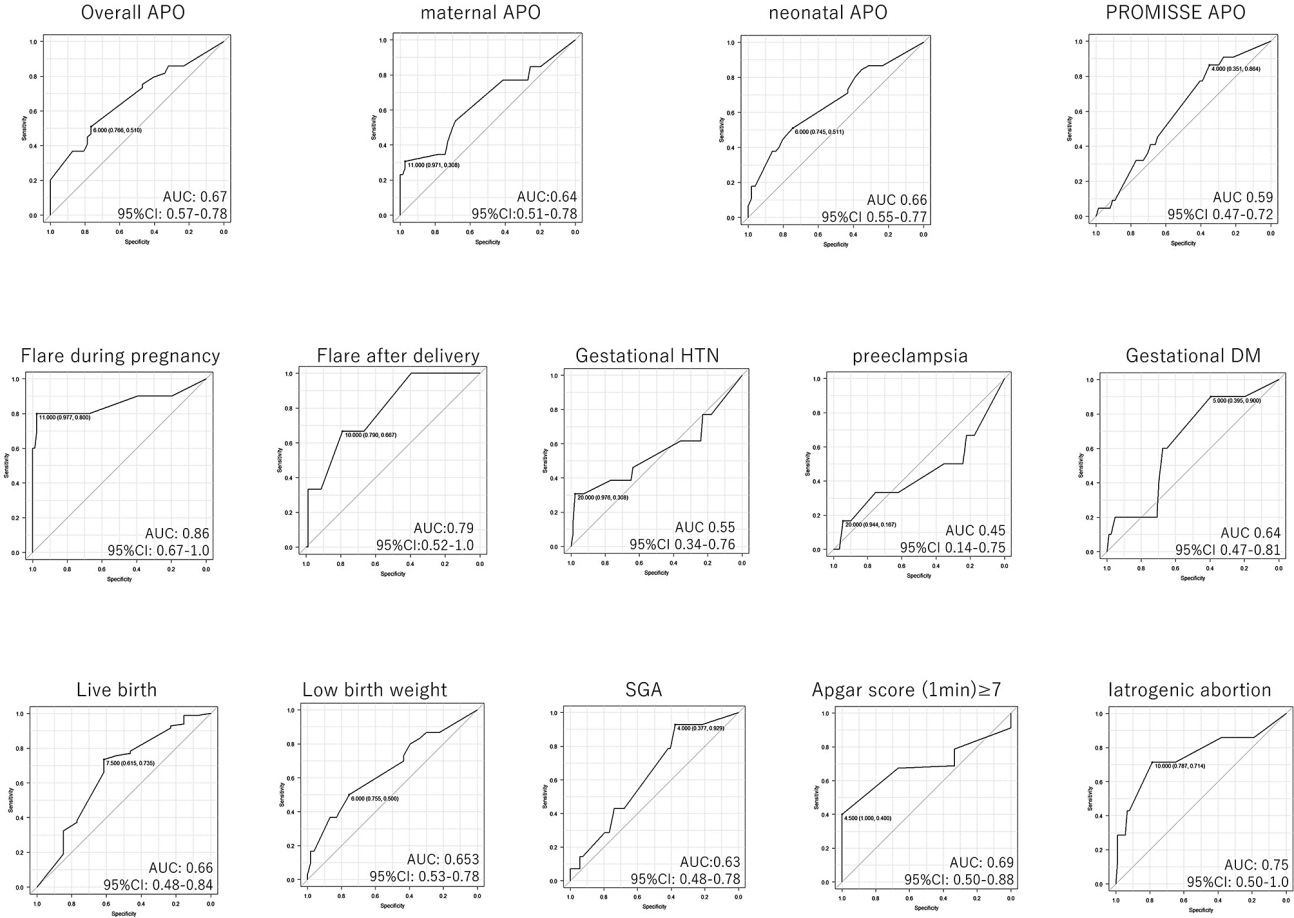
ROC curve for glucocorticoid dosage at conception to predict each APO ROC, Receiver operating characteristic; APO, adverse pregnancy outcome

We constructed ROC curves to determine the optimal cut-off values for predicting each APO based on the glucocorticoid dosage at conception. As depicted in Fig. 1, a PSL dosage of ≥ 6 mg/day at conception was associated with an increased risk of overall APO and neonatal APO. PSL dosage of ≥ 7.5 mg/day at conception was associated with a decreased rate of live birth (overall APO: AUC 0.67, 95%CI 0.57–0.78 sensitivity 0.51, specificity 0.77; neonatal APO: AUC 0.66, 95%CI 0.55–0.77 sensitivity 0.5 specificity 0.74; live birth: AUC 0.66, 95%CI 0.48–0.84, sensitivity 0.73, specificity 0.62).

In addition, PSL dosages of ≥ 11 mg/day and ≥ 10 mg/day at conception were associated with an increased flare rate during pregnancy and after delivery, respectively (flare during pregnancy: AUC 0.86, 95%CI 0.67–1.0 sensitivity 0.80 specificity 0.98; flare after delivery: AUC 0.79, 95%CI 0.52–1.0 sensitivity 0.67, specificity 0.79).

Furthermore, we divided the patients into two groups: those with a PSL dosage of ≥ 7.5 mg/day at conception (*n* = 32) and those with a PSL dosage of < 7.5 mg/day at conception (*n* = 64). Our analysis revealed that the risk of overall APO and neonatal APO was statistically higher in the group with a PSL dosage of ≥ 7.5 mg/day compared with the group with a PSL dosage of < 7.5 mg/day (overall APO: OR 3.01, 95%CI 1.23–7.39, *p* = 0.016; neonatal APO: OR 2.98, 95%CI 1.23–7.22, *p* = 0.016). (Table 5 and Supplementary Tables S9 and S10).

**Table 5:** Glucocorticoid dosage (prednisolone equivalent) ≥ 7.5 mg/day at conception and risk of APO

Factor	Glucocorticoid dosage at conception			Logistic regression model Univariate analysis			Logistic regression model multivariate analysis		
	PSL < 7.5 mg/day	PSL ≥ 7.5 mg/day	*p*-value	OR^a^	95%CI	*P* value	aOR^b^	95%CI	*P* value
*n*	64	32							
**overall APO (%)**	**27 (42.2)**	**22 (68.8)**	**0.018**	**3.01**	**1.23–7.39**	**0.016**	**3.11**	**1.20–8.04**	**0.019**
Maternal APO (%)	14 (21.9)	12 (37.5)	0.14	2.14	0.85–5.43	0.11	2.78	0.98–7.88	0.055
**Neonatal APO (%)**	**25 (39.1)**	**21 (65.6)**	**0.018**	**2.98**	**1.23–7.22**	**0.016**	**2.91**	**1.14–7.38**	**0.025**
PROMISSE APO (%)	13 (20.3)	9 (28.1)	0.44	1.54	0.58–4.10	0.39	1.59	0.56–4.50	0.38
**Flare during pregnancy (%)**	**2 (3.1)**	**8 (25.0)**		**10.3**	**2.05–42.2**				
Flare after delivery (%)	1 (1.7)	2 (8.0)		5.04	0.44–58.3				
Gestational DM (%)	5 (7.8)	5 (15.6)		2.19	0.58–8.19				
Preeclampsia (%)	4 (6.2)	2 (6.2)		1.0	0.17–5.77				
Hypertensive disorders in pregnancy (%)	8 (12.5)	5 (15.6)		1.3	0.39–4.34				
HELLP syndrome (%)	1 (1.6)	1 (3.1)		2.03	0.12–33.6				
Oligohydramnios (%)	2 (3.2)	6 (18.8)		7.04	1.33–37.2				
Live birth (%)	59 (92.2)	24 (75.0)		0.25	0.08–0.86				
Total duration of gestation (days)	268.0 [262.0, 276.0]	261.00 [234.8, 269.5]		NA	NA				
Preterm birth (%)	9 (15.3)	5 (17.9)		1.21	0.36–1.0				
Spontaneous abortion (%)	2 (3.2)	1 (3.2)		1.02	0.09–11.7				
Missed abortion (%)	1 (1.6)	3 (9.4)		6.31	0.63–63.3				
Iatrogenic abortion (%)	2 (3.1)	5 (15.6)		5.74	1.05–31.5				
Still birth (%)	0 (0.0)	0 (0.0)		NA	NA				
Height at birth (cm)	47.9 [46.2, 49.4]	46.2 [44.1, 48.1]		NA	NA				
Weight at birth (g)	2732.0 [2461.0, 3014.5]	2421.0 [2168.0, 2825.8]		NA	NA				
Low birth weight (%)	17 (28.8)	13 (54.2)		2.92	1.09–7.79				
SGA (%)	8 (13.6)	6 (25.0)		2.13	0.65–6.96				
Apgar score (1 m)	8.00 [8.00, 8.00]	8.00 [8.00, 8.25]		NA	NA				
Apgar Score (5 m)	9.00 [9.00, 9.00]	9.00 [9.00, 9.00]		NA	NA				
Apgar.score.1 m > 7 (%)	57 (96.6)	23 (95.8)		0.81	0.07–9.34				
Apgar.score.5 m > 7 (%)	59 (100.0)	24 (100.0)		NA	NA				
Major malformation (%)	2 (3.4)	0 (0.0)		NA	NA				
Death of neonate	0 (0.0)	0 (0.0)		NA	NA				

a Odds ratio of glucocorticoid dosage (prednisolone equivalent) ≥ 7.5 mg/day for each APO

b adjusted odds ratio of glucocorticoid dosage (prednisolone equivalent) ≥ 7.5 mg/day for each APO

※multivariate analysis adjusted for renal manifestation, hydroxychloroquine prescription, and aspirin prescription at conception

APO; adverse pregnancy outcome, DM; diabetes mellitus, NA; not applicable, OR; odds ratio, PROMISSE; Predictors of Pregnancy Outcome: Biomarkers in Antiphospholipid Antibody Syndrome and Systemic Lupus Erythematosus, SGA; small for gestational age

In addition, as shown in Table 5 and supplementary Table S11, multivariate analysis also showed increase in overall/maternal/neonatal/PROMISSE APO ratio in those treated with PSL ≥ 7.5 mg/day (overall APO: aOR 3.11, 95%CI 1.20–8.04, *p* = 0.019, maternal APO: aOR 2.78, 95%CI 0.98–7.88, *p* = 0.055, neonatal APO: aOR 2.91, 95%CI 1.14–7.38, *p* = 0.025, PROMISSE APO: aOR 1.59, 95%CI 0.56–4.50, *p* = 0.38).

## Discussion

In this multicenter retrospective cohort study, we have demonstrated that achieving Zen/Doria remission is associated with a reduction in the APO ratio, including flare rate during pregnancy, iatrogenic abortion, and low birth weight. In addition, the live birth rate was higher in patients with remission than those without.

Previous reports have indicated the frequency of each specific APO in pregnant patients with SLE as follows: flare during pregnancy (21.4–64%), hypertensive disorders of pregnancy (0.99–45%), gestational diabetes mellitus (0–11%), preeclampsia (5.4–20.2%), HELLP syndrome (0.3–0.66%), preterm birth (9–56%), spontaneous abortion (0.4–25%), SGA (10–28.5%), Apgar score < 7 at 1 min (1–18%) [36], and PROMISSE APO (approximately 19%) [20]. Therefore, the APO ratio in our cohort aligns with the data reported in these previous studies. Kim et al. reported that achieving LLDAS was associated with a reduced risk of maternal and neonatal APOs (maternal APO: OR 0.18, 95%CI 0.04–0.74, *p* = 0.016; neonatal APO: OR 0.21, 95%CI 0.06–0.65, *p* = 0.01) [37]. Furthermore, Chiara et al. and Ntali et al. demonstrated that achieving DORIS remission was associated with a decreased APO ratio [5, 38].

These findings are consistent with our data on Zen/Doria remission. One strength of our study is that we assessed the risk of specific APOs based on different remission definitions: clinical remission on corticosteroids, clinical remission off corticosteroids, and complete remission.

Moreover, previous reports have indicated that serological activity in the overall SLE population is associated with worse pregnancy outcomes [39–41]; however, no study has specifically addressed the impact of a serologically active state on pregnancy outcomes in patients with SLE in clinical remission.

A questionnaire-based investigation revealed that 20–30% of clinicians prohibit pregnancy in SLE patients in clinical remission with mildly active serology [42]. Our study confirmed no increased risk of APOs between pregnant patients in clinical remission with active serology and those in both serologically and clinically stable pregnant patients. Achieving a serologically and clinically stable state is ideal in SLE management to reduce the risk of flares [6, 7]; however, it often takes considerable time to achieve this state and can be particularly challenging in patients planning pregnancy, given the limited therapeutic options compatible with gestation as medication alterations or discontinuations may be necessary.

Our findings will encourage patients with SLE to consider motherhood and physicians to support their patients’ pregnancy plans, as attaining clinical remission with active serology is generally more feasible than achieving both clinical and serological remission.

Furthermore, we discovered that the risk of overall and neonatal APOs could be reduced by lowering the glucocorticoid dosage to 6 mg/day while using pregnancy-compatible medications. In addition, reducing the glucocorticoid dosage to 7.5 mg/day can improve the live birth rate. These results align with those of previous studies demonstrating an association between glucocorticoid use and APOs, including preterm birth and low birth weight [43–46].

Consistent with earlier reports, our findings also revealed that glucocorticoid dosages exceeding 7.5 mg/day were associated with an increased risk of overall/neonatal APO, a shorter gestational period, and lower birth weight.

Advancements in SLE care have improved pregnancy outcomes; however, the APO ratio remains higher in patients with SLE than in the general population [47]. Our results highlight that further improvements in pregnancy outcomes can be achieved by attaining clinical remission and reducing the glucocorticoid dosage to below 7.5 mg/day whenever feasible.

### Limitation

Our study has few limitations. First, the number of participants was limited, potentially affecting our ability to evaluate less frequent types of APOs, so future studies with larger cohort are needed to validate our findings. Second, the cohort predominantly included Japanese pregnant patients, potentially limiting the applicability of our findings to diverse patient demographics. Third, we excluded patients who delivered at clinics or institutions other than our center. As both of our centers are tertiary teaching hospitals catering to patients with relatively acute conditions, the severity of pregnancy cases tended to be heightened, which might have influenced the APO ratio.

Fourth, long-term follow-up data on the offspring of pregnant women with SLE were lacking. Considering reports indicating an augmented risk of neurodevelopmental disorders in children born to mothers with SLE [48], assessing the long-term effects of pregnancy outcomes on remission status in future cohort studies is imperative.

Finally, hydroxychloroquine and aspirin treatments were administered to a relatively modest population, likely because of the delayed approval of hydroxychloroquine in Japan in October 2015, with 40/96 (41.6%) deliveries occurring before the approval of hydroxychloroquine in the nation. Furthermore, half of the pregnancies (49/96) happened before the publication of the article discussing aspirin’s role in preventing preeclampsia in those pregnant individuals exhibiting an elevated risk for preeclampsia. (August 2017) [24].

## Conclusion

We demonstrated that achieving remission is associated with a decrease in the APO ratio and an increase in the live birth rate. Achieving clinical and serological remission is the optimal goal in SLE management; however, it is not always necessary to target serological negativity to improve pregnancy outcomes in patients with SLE who are already in clinical remission, particularly in cases of advanced maternal age which necessitates urgent pregnancy planning. Furthermore, it is recommended to aim for a glucocorticoid dose < 7.5 mg/day (PSL equivalent) before pregnancy to reduce the likelihood of APOs.

### Electronic supplementary material

Below is the link to the electronic supplementary material.

Supplementary Material 1

## Data Availability

All data generated/analyzed during this study, and supplementary data are included in this article.

## Abbreviations

ACR: American College of Rheumatology
aOR: Adjusted odds ratio
APO: Adverse pregnancy outcomes
AUC: Area under the curve
BILAG: British Isles Lupus Assessment Group
BMI: Body mass index
CI: Confidence interval
DORIS: Definitions of Remission in SLE
EULAR: European League Against Rheumatism
OR: Odds ratio
PROMISSE: Predictors of Pregnancy Outcome: Biomarkers in Antiphospholipid Antibody Syndrome and SLE
PSL: Prednisolone
ROC curve: Receiver Operating Characteristic curve
SGA: Small for gestational age
SLE: Systemic lupus erythematosus
LLDAS: Lupus low disease activity state
