# Supplementary material for: Effect of remission, clinical remission with active serology, and glucocorticoid dosage on the pregnancy outcome of pregnant patients with systemic lupus erythematosus
Source: Arthritis Res Ther. 2024 Mar 9;26:63. doi: 10.1186/s13075-024-03298-6 (PMC10924338; doi:10.1186/s13075-024-03298-6)
Supplement: Supplementary file 1 — Supplementary Material 1 [file 13075_2024_3298_MOESM1_ESM.docx]

**Supplementary Figure S1**: Patient flow chart

**
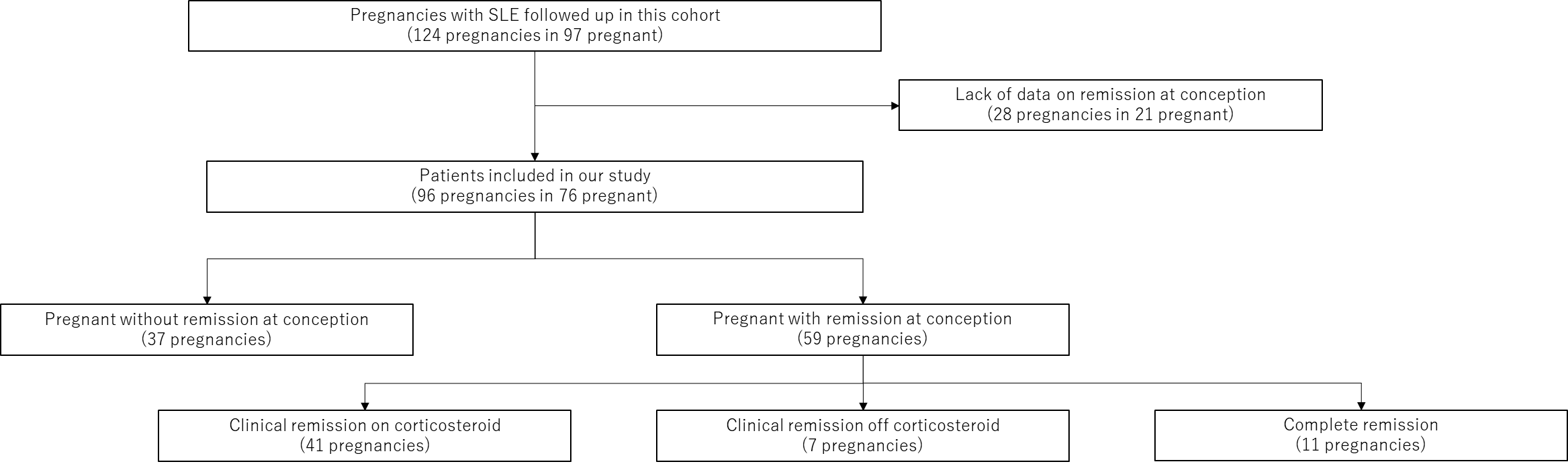
**

**Supplementary Table S1. Baseline characteristics according to the attainment of detailed definition of remission at conception**

|  | Zen/Doria remission | | | | | |
| --- | --- | --- | --- | --- | --- | --- |
| Factor | No remission | clinical remission on CS | clinical remission off CS | complete remission | p-value |  |
| n | 37 | 41 | 7 | 11 |  |  |
| ***Epidemiological findings*** |  |  |  |  |  |  |
| Age at conception (years old) | 34.0 [31.0, 36.0] | 33.0 [29.0, 36.0] | 29.0 [27.0, 32.5] | 33.0 [31.5, 35.5] | 0.11 |  |
| BMI | 19.7 [19.0, 21.3] | 20.2 [18.2, 21.6] | 19.7 [17.7, 20.1] | 19.3 [17.8, 20.5] | 0.55 |  |
| **Duration of SLE (days)** | **2943 [1893, 5198]** | **2965 [2270, 5464]** | **1552 [1212, 2126]** | **1476 [1152, 2165]** | **<0.01** |  |
| Smoking history (%) | 3 (8.1) | 3 (7.3) | 0 (0.0) | 1 (9.1) | 0.89 |  |
| Previous spontaneous abortion (%) | 6 (16.2) | 9 (22.0) | 0 (0.0) | 3 (27.3) | 0.46 |  |
| Previous Anti-hypertensive med use (%) | 4 (10.8) | 2 (4.9) | 0 (0.0) | 0 (0.0) | 0.45 |  |
| Multiparous (%) | 15 (40.5) | 17 (42.5) | 3 (42.9) | 1 (9.1) | 0.22 |  |
| Infertility treatment (%) | 10 (27.0) | 10 (24.4) | 2 (28.6) | 5 (45.5) | 0.59 |  |
| **Any flare at conception** (%) | **6 (16.2)** | **0 (0.0)** | **0 (0.0)** | **0 (0.0)** | **0.017** |  |
| ***Organ manifestation*** |  |  |  |  |  |  |
| Joint/muscular manifestation (%) | 26 (70.3) | 26 (63.4) | 4 (57.1) | 8 (72.7) | 0.83 |  |
| Skin/mucocutaneous manifestation (%) | 27 (73.0) | 32 (78.0) | 5 (71.4) | 7 (63.6) | 0.80 |  |
| Renal manifestation (%) | 13 (35.1) | 10 (24.4) | 0 (0.0) | 2 (18.2) | 0.22 |  |
| Lupus nephritis class III/IV (%) | 5 (13.5) | 4 (9.8) | 0 (0.0) | 0 (0.0) | 0.46 |  |
| **Serositis** (%) | **7 (18.9)** | **15 (36.6)** | **0 (0.0)** | **0 (0.0)** | **0.018** |  |
| Neurological manifestation (%) | 2 (5.4) | 5 (12.2) | 0 (0.0) | 2 (18.2) | 0.42 |  |
| Hematological manifestation (%) | 28 (75.7) | 37 (90.2) | 7 (100.0) | 9 (81.8) | 0.21 |  |
| ***Immunological profile*** |  |  |  |  |  |  |
| **Anti-dsDNA antibody** (%) | **19 (51.4)** | **34 (82.9)** | **6 (85.7)** | **4 (36.4)** | **<0.01** |  |
| Anti-RNP antibody (%) | 12 (48.0) | 13 (38.2) | 1 (20.0) | 4 (40.0) | 0.68 |  |
| Anti-Sm antibody (%) | 8 (25.8) | 19 (48.7) | 2 (28.6) | 2 (18.2) | 0.12 |  |
| **Anti-SSA antibody** (%) | **21 (56.8)** | **32 (78.0)** | **4 (66.7)** | **4 (36.4)** | **0.045** |  |
| Anti-SSB antibody (%) | 2 (7.7) | 8 (22.9) | 0 (0.0) | 2 (25.0) | 0.29 |  |
| LAC (%) | 4 (11.1) | 6 (15.0) | 1 (16.7) | 1 (9.1) | 0.92 |  |
| **Anti-CL** antibody (%) | **5 (15.2)** | **8 (20.0)** | **4 (57.1)** | **5 (45.5)** | **0.033** |  |
| Anti-CLβ2GPI antibody (%) | 3 (8.3) | 4 (9.8) | 0 (0.0) | 1 (9.1) | 0.89 |  |
| Low C3 (%) | 23 (65.7) | 31 (75.6) | 4 (57.1) | 5 (45.5) | 0.26 |  |
| Low C4 (%) | 30 (85.7) | 34 (82.9) | 5 (71.4) | 8 (72.7) | 0.68 |  |

Ab: antibody; BMI: body mass index; CL: cardiolipin; CS: corticosteroid; LAC: lupus anticoagulant; SLE: systemic lupus erythematosus

**Supplementary Table S2: Treatment regimen at each time point of pregnancy**

|  | 6–3 m prior | | | 3–0 m prior | | | First trimester | | | second trimester | | | Third trimester | | | 0–3m | | | 3–6m | | |
| --- | --- | --- | --- | --- | --- | --- | --- | --- | --- | --- | --- | --- | --- | --- | --- | --- | --- | --- | --- | --- | --- |
|  | Remission achievement  at conception | | | Remission achievement  at conception | | | Remission achievement  at conception | | | Remission achievement  at conception | | | Remission achievement  at conception | | | Remission achievement  at conception | | | Remission achievement  at conception | | |
| Factor | (-) | (+) | p-value | (-) | (+) | p-value | (-) | (+) | p-value | (-) | (+) | p-value | (-) | (+) | p-value | (-) | (+) | p-value | (-) | (+) | p-value |
| n | 31 | 53 |  | 31 | 53 |  | 37 | 59 |  | 37 | 57 |  | 30 | 56 |  | 32 | 54 |  | 30 | 50 |  |
| PSL | **10.00 [6.00, 10.00]** | **4.00 [1.00, 5.00]** | **<0.01** | **10.00 [7.00, 10.00]** | **4.00 [0.00, 5.00]** | **<0.01** | **10.00 [8.00, 11.00]** | **4.00 [0.00, 5.00]** | **<0.01** | **10.00 [8.00, 10.00]** | **4.25 [0.00, 5.00]** | **<0.01** | **10.00 [8.00, 10.00]** | **4.75 [0.00, 5.00]** | **<0.01** | **10.00 [9.12, 10.50]** | **5.00 [0.25, 9.75]** | **<0.01** | **9.25 [7.50, 10.00]** | **4.75 [1.12, 5.00]** | **<0.01** |
| HCQ | **10 (27.8)** | **29 (50.9)** | **0.033** | **11 (30.6)** | **31 (54.4)** | **0.033** | 14 (37.8) | 32 (54.2) | 0.14 | 12 (36.4) | 32 (57.1) | 0.079 | **10 (33.3)** | **31 (57.4)** | **0.042** | **10 (31.2)** | **31 (57.4)** | **0.026** | **10 (31.2)** | **30 (58.8)** | **0.023** |
| Tac. | 12 (38.7) | 13 (24.5) | 0.22 | 12 (38.7) | 13 (24.5) | 0.22 | 13 (35.1) | 13 (22.0) | 0.24 | 12 (36.4) | 11 (19.6) | 0.13 | 9 (30.0) | 9 (16.7) | 0.17 | 11 (34.4) | 10 (18.5) | 0.12 | 12 (40.0) | 10 (20.4) | 0.074 |
| CyA | 0 (0.0) | 2 (3.8) | 0.53 | 0 (0.0) | 2 (3.8) | 0.53 | 0 (0.0) | 2 (3.4) | 0.52 | 0 (0.0) | 2 (3.6) | 0.53 | 0 (0.0) | 2 (3.7) | 0.54 | 0 (0.0) | 2 (3.7) | 0.53 | 0 (0.0) | 2 (4.1) | 0.52 |
| AZA | 2 (6.5) | 6 (11.3) | 0.70 | 2 (6.5) | 5 (9.4) | 1.0 | 2 (5.4) | 4 (6.8) | 1.00 | 2 (6.1) | 3 (5.4) | 1.00 | 2 (6.7) | 3 (5.6) | 1.00 | 1 (3.1) | 2 (3.7) | 1.00 | 1 (3.3) | 3 (6.1) | 1.00 |
| MMF | 1 (3.2) | 1 (1.9) | 1.00 | 0 (0.0) | 0 (0.0) | NA | 1 (2.7) | 0 (0.0) | 0.39 | 0 (0.0) | 0 (0.0) | NA | 0 (0.0) | 0 (0.0) | NA | 0 (0.0) | 0 (0.0) | NA | 0 (0.0) | 0 (0.0) | NA |
| MZR | 0 (0.0) | 0 (0.0) | NA | 0 (0.0) | 1 (1.9) | 1.0 | 0 (0.0) | 1 (1.7) | 1.00 | 0 (0.0) | 0 (0.0) | NA | 0 (0.0) | 0 (0.0) | NA | 0 (0.0) | 0 (0.0) | 1.00 | 0 (0.0) | 0 (0.0) | 1.00 |
| MTX | 0 (0.0) | 0 (0.0) | NA | 0 (0.0) | 0 (0.0) | NA | 0 (0.0) | 0 (0.0) | NA | 0 (0.0) | 0 (0.0) | NA | 0 (0.0) | 0 (0.0) | NA | 0 (0.0) | 0 (0.0) | NA | 0 (0.0) | 0 (0.0) | NA |
| BEL | 2 (6.5) | 2 (3.8) | 0.62 | 2 (6.5) | 1 (1.9) | 0.55 | 2 (5.4) | 1 (1.7) | 0.56 | 0 (0.0) | 0 (0.0) | NA | 0 (0.0) | 0 (0.0) | NA | 0 (0.0) | 1 (1.9) | 1.00 | 0 (0.0) | 1 (2.0) | 1.00 |
| CY/RTX/plasma exchange | 0 (0.0) | 0 (0.0) | NA | 0 (0.0) | 0 (0.0) | NA | 0 (0.0) | 0 (0.0) | NA | 0 (0.0) | 0 (0.0) | NA | 0 (0.0) | 0 (0.0) | NA | 0 (0.0) | 0 (0.0) | NA | 0 (0.0) | 0 (0.0) | NA |
| IVIg | 0 (0.0) | 0 (0.0) | NA | 0 (0.0) | 0 (0.0) | NA | 0 (0.0) | 0 (0.0) | NA | 0 (0.0) | 0 (0.0) | NA | 1 (3.3) | 0 (0.0) | 0.36 | 1 (3.2) | 0 (0.0) | 0.37 | 0 (0.0) | 0 (0.0) | NA |
| aspirin | 1 (3.2) | 9 (17.0) | 0.083 | 2 (6.5) | 10 (18.9) | 0.20 | 12 (32.4) | 30 (50.8) | 0.093 | 13 (39.4) | 32 (58.2) | 0.12 | 9 (30.0) | 21 (39.6) | 0.48 |  |  |  |  |  |  |

AZA: azathioprine, BEL: belimumab, CY: cyclophosphamide, CyA: cyclosporine, GC: glucocorticoid, HCQ: hydroxychloroquine, IVIg: intravenous immunoglobulin, MMF: mycophenolate mofetil, MTX: methotrexate, MZR: mizoribine, NA: not available, PE: plasma exchange, PSL: prednisolone, RTX: rituximab, Tac: tacrolimus

**Supplementary Table S3. Treatment regimen at conception according to the detailed definition of remission**

|  | Zen/Doria remission | | | | |
| --- | --- | --- | --- | --- | --- |
| Factor | No remission | Clinical remission on CS | Clinical remission off CS | Complete remission | p-value |
| n | 37 | 41 | 7 | 11 |  |
| **GC (mg/day)** | **10.00 [8.00, 11.00]** | **5.00 [3.00, 5.00]** | **0.00 [0.00, 0.00]** | **0.00 [0.00, 0.00]** | **<0.01** |
| HCQ (%) | 14 (37.8) | 25 (61.0) | 4 (57.1) | 3 (27.3) | 0.093 |
| **Tac (%)** | **13 (35.1)** | **13 (31.7)** | **0 (0.0)** | **0 (0.0)** | **0.039** |
| CyA (%) | 0 (0.0) | 2 (4.9) | 0 (0.0) | 0 (0.0) | 0.43 |
| AZA (%) | 2 (5.4) | 4 (9.8) | 0 (0.0) | 0 (0.0) | 0.55 |
| MMF (%) | 1 (2.7) | 0 (0.0) | 0 (0.0) | 0 (0.0) | 0.66 |
| MZR (%) | 0 (0.0) | 1 (2.4) | 0 (0.0) | 0 (0.0) | 0.72 |
| MTX (%) | 0 (0.0) | 0 (0.0) | 0 (0.0) | 0 (0.0) | NA |
| BEL (%) | 2 (5.4) | 1 (2.4) | 0 (0.0) | 0 (0.0) | 0.73 |
| RTX/CY/PE/IVIg (%) | 0 (0.0) | 0 (0.0) | 0 (0.0) | 0 (0.0) | NA |
| **aspirin (%)** | 12 (32.4) | 21 (51.2) | 3 (42.9) | 6 (54.5) | 0.34 |

AZA: azathioprine, BEL: belimumab, CS: corticosteroid, CY: cyclophosphamide, CyA: cyclosporine, HCQ: hydroxychloroquine, IVIg: intravenous immunoglobulin, MMF: mycophenolate mofetil, MTX: methotrexate, MZR: mizoribine, NA: not available, PE: plasma exchange, PSL: prednisolone, RTX: rituximab, Tac: tacrolimus

**Supplementary table S4.** **multivariate analysis on risk of averse pregnancy outcome according to the achievement of remission at conception**

|  | Multivariate logistic regression model  analysis 1 | | | Multivariate logistic regression model  analysis 2 | | | Multivariate logistic regression model  analysis 3 | | | Multivariate logistic regression model  analysis 4 | | | Multivariate logistic regression model  analysis 5 | | |
| --- | --- | --- | --- | --- | --- | --- | --- | --- | --- | --- | --- | --- | --- | --- | --- |
| Factor | aOR^a^ | 95% CI | p value | aOR^a^ | 95% CI | p value | aOR^a^ | 95% CI | p value | aOR^a^ | 95% CI | p value | aOR^a^ | 95% CI | p value |
| **Overall APO (%)** | **0.28** | **0.11–0.70** | **<0.01** | **0.33** | **0.13–0.83** | **0.019** | **0.31** | **0.12-0.79** | **0.014** | 0.51 | 0.18-1.39 | 0.19 | 0.59 | 0.21-1.66 | 0.32 |
| **Maternal APO (%)** | **0.33** | **0.12–090** | **0.030** | 0.38 | 0.13–1.06 | 0.065 | 0.41 | 0.15-1.15 | 0.090 | 0.73 | 0.23-2.37 | 0.60 | 0.95 | 0.28-3.26 | 0.94 |
| **Neonatal APO (%)** | **0.37** | **0.15–0.90** | **0.029** | **0.38** | **0.14–0.98** | **0.046** | **0.35** | **0.14-0.88** | **0.026** | 0.53 | 0.20-1.43 | 0.21 | 0.56 | 0.20-1.58 | 0.27 |
| PROMISSE APO (%) | 0.64 | 0.23–1.76 | 0.38 | 0.67 | 0.24–1.89 | 0.45 | 0.57 | 0.20-1.61 | 0.29 | 0.51 | 0.16-1.58 | 0.24 | 0.50 | 0.16-1.59 | 0.24 |

a adjusted odds ratio of Zen/Doria remission attainment for each APO

analysis 1: adjusted for renal manifestation, hydroxychloroquine prescription, and aspirin prescription at conception.

analysis 2: adjusted for renal manifestation, hydroxychloroquine prescription, aspirin prescription at conception, and LAC positivity

analysis 3: adjusted for renal manifestation, hydroxychloroquine prescription, aspirin prescription at conception, and thrombocytopenia at conception

analysis 4^※^: adjusted for renal manifestation, hydroxychloroquine prescription, aspirin prescription at conception, and pregnancy planning

analysis 5^※^: adjusted for renal manifestation, hydroxychloroquine prescription, aspirin prescription at conception, and LAC positivity, thrombocytopenia at conception and pregnancy planning

aOR; adjusted odds ratio APO; adverse pregnancy outcome, CI; confidence interval, LAC; lupus anticoagulant, PROMISSE; Predictors of Pregnancy Outcome: Biomarkers in Antiphospholipid Antibody Syndrome and Systemic Lupus Erythematosus, SGA; small for gestational age

※SLE disease stability is one of the factors to allow patients to plan pregnancy. Given this context, we consider 'planned pregnancy' as a potential mediator in the relationship between remission and APOs, rather than a confounder.

**Supplementary Table S5. Prevalence of each adverse pregnancy outcome according to the attainment of detailed definition of remission at conception**

|  | Zen/Doria remission | | | | |
| --- | --- | --- | --- | --- | --- |
| Factor | No remission | Clinical remission on CS | Clinical remission off CS | Complete remission | p-value |
| n | 37 | 41 | 7 | 11 |  |
| **Overall APO (%)** | **26 (70.3)** | **17 (41.5)** | **2 (28.6)** | **4 (36.4)** | **0.025** |
| **Maternal APO (%)** | **15 (40.5)** | **8 (19.5)** | **0 (0.0)** | **3 (27.3)** | **0.066** |
| Neonatal APO (%) | 23 (62.2) | 17 (41.5) | 2 (28.6) | 4 (36.4) | 0.15 |
| PROMISSE APO (%) | 10 (27.0) | 10 (24.4) | 1 (14.3) | 1 (9.1) | 0.60 |
| Flare during pregnancy (%) | 8 (21.6) | 1 (2.4) | 0 (0.0) | 1 (9.1) |  |
| Flare after delivery (%) | 2 (6.7) | 1 (2.6) | 0 (0.0) | 0 (0.0) |  |
| Gestational DM (%) | 6 (16.2) | 3 (7.3) | 0 (0.0) | 1 (9.1) |  |
| Preeclampsia (%) | 3 (8.1) | 2 (4.9) | 0 (0.0) | 1 (9.1) |  |
| hypertensive disorder of pregnancy (%) | 7 (18.9) | 4 (9.8) | 0 (0.0) | 2 (18.2) |  |
| HELLP syndrome (%) | 1 (2.7) | 1 (2.4) | 0 (0.0) | 0 (0.0) |  |
| Oligohydramnios (%) | 6 (16.2) | 1 (2.4) | 1 (16.7) | 0 (0.0) |  |
| Maternal death (%) | 0 (0.0) | 0 (0.0) | 0 (0.0) | 0 (0.0) |  |
| Live birth (%) | 29 (78.4) | 38 (92.7) | 7 (100.0) | 9 (81.8) |  |
| Total duration of gestation (days) | 262.0 [242.0, 271.0] | 267.0 [262.0, 275.0] | 276.0 [273.5, 278.5] | 270.0 [253.0, 281.5] |  |
| Preterm birth (%) | 6 (18.2) | 6 (15.8) | 1 (14.3) | 1 (11.1) |  |
| Spontaneous abortion (%) | 1 (2.8) | 2 (5.0) | 0 (0.0) | 0 (0.0) |  |
| Missed abortion (%) | 3 (8.1) | 0 (0.0) | 0 (0.0) | 1 (9.1) |  |
| Iatrogenic abortion (%) | 5 (13.5) | 1 (2.4) | 0 (0.0) | 1 (9.1) |  |
| Stillbirth (%) | 0 (0.0) | 0 (0.0) | 0 (0.0) | 0 (0.0) |  |
| Height at birth (cm) | 46.0 [43.8, 48.0] | 47.4 [46.0, 49.0] | 49.1 [48.5, 49.5] | 49.00 [47.00, 50.00] |  |
| Weight at birth (g) | 2472 [2202, 2896] | 2658 [2452, 2925] | 3120 [2727, 3230] | 3004 [2574, 3176] |  |
| Low birth weight (%) | 15 (51.7) | 11 (28.9) | 2 (28.6) | 2 (22.2) |  |
| SGA (%) | 6 (20.7) | 7 (18.4) | 1 (14.3) | 0 (0.0) |  |
| Apgar score (1m) | 8.0 [8.00, 8.00] | 8.00 [8.00, 8.00] | 8.00 [8.00, 8.00] | 8.00 [8.00, 8.00] |  |
| Apgar Score (5m) | 9.00 [9.00, 9.00] | 9.00 [9.00, 9.00] | 9.00 [8.50, 9.00] | 9.00 [9.00, 9.00] |  |
| Apgar.score.1m>7 (%) | 27 (93.1) | 37 (97.4) | 7 (100.0) | 9 (100.0) |  |
| Apgar.score.5m>7 (%) | 29 (100.0) | 38 (100.0) | 7 (100.0) | 9 (100.0) |  |
| Major malformation (%) | 1 (3.4) | 1 (2.6) | 0 (0.0) | 0 (0.0) |  |
| Death of neonate (%) | 0 (0.0) | 0 (0.0) | 0 (0.0) | 0 (0.0) |  |

APO, adverse pregnancy outcome; CS, corticosteroid; DM, diabetes mellitus; HELLP, hemolysis; elevated liver enzymes and low platelets; NA, not available; OR, odds ratio; PROMISSE, Predictors of Pregnancy Outcome: Biomarkers in Antiphospholipid Antibody Syndrome and Systemic Lupus Erythematosus; SGA, small for gestational age.

**Supplementary Table S6. Odds ratio of each adverse pregnancy outcome according to the attainment of detailed definition of remission at conception**

|  | Logistic regression model | | |
| --- | --- | --- | --- |
| Factor | OR | 95%CI | P-value |
| ***overall APO*** |  |  |  |
| No remission | Ref | Ref | Ref |
| **Clinical remission on CS** | **0.3** | **0.12–0.77** | **0.012** |
| Clinical remission off CS | 0.17 | 0.03–1.01 | 0.051 |
| **Complete remission** | **0.24** | **0.06–1.00** | **0.049** |
| **Any remission** | **0.27** | **0.11–0.65** | **<0.01** |
| ***Maternal APO*** |  |  |  |
| No remission | Ref | Ref | Ref |
| **Clinical remission on CS** | **0.36** | **0.13–0.98** | **0.045** |
| Clinical remission off CS | NA | NA | NA |
| Complete remission | 0.55 | 0.13–2.42 | 0.43 |
| **Any remission** | **0.34** | **0.13–0.85** | **0.021** |
| ***Neonatal APO*** |  |  |  |
| No remission | Ref | Ref | Ref |
| Clinical remission on CS | 0.43 | 0.17–1.07 | 0.070 |
| Clinical remission off CS | 0.24 | 0.04–1.43 | 0.12 |
| Complete remission | 0.35 | 0.09–1.41 | 0.14 |
| **Any remission** | **0.39** | **0.17–0.90** | **0.028** |
| ***PROMISSE APO*** |  |  |  |
| No remission | Ref | Ref | Ref |
| Clinical remission on CS | 0.87 | 0.32–2.41 | 0.79 |
| Clinical remission off CS | 0.45 | 0.05–4.22 | 0.48 |
| Complete remission | 0.27 | 0.03–2.39 | 0.23 |
| Any remission | 0.69 | 0.26–1.81 | 0.45 |
| ***Flare during pregnancy*** |  |  |  |
| No remission | Ref | Ref |  |
| Clinical remission on CS | 0.91 | 0.01–0.77 |  |
| Clinical remission off CS | NA | NA |  |
| Complete remission | 0.363 | 0.04–3.27 |  |
| Any remission | 0.13 | 0.03–0.64 |  |
| ***Flare after delivery*** |  |  |  |
| No remission | Ref | Ref |  |
| Clinical remission on CS | 0.38 | 0.03–4.39 |  |
| Clinical remission off CS | NA | NA |  |
| Complete remission | NA | NA |  |
| Any remission | 0.26 | 0.23–3.04 |  |
| ***gestational diabetes mellitus*** |  |  |  |
| No remission | Ref | Ref |  |
| Clinical remission on CS | 0.41 | 0.943–1.76 |  |
| Clinical remission off CS | NA | NA |  |
| Complete remission | 0.41 | 0.09–1.76 |  |
| Any remission | 0.38 | 0.10–1.43 |  |
| ***Preeclampsia*** |  |  |  |
| No remission | Ref | Ref |  |
| Clinical remission on CS | 0.58 | 0.09–3.69 |  |
| Clinical remission off CS | NA | NA |  |
| Complete remission | 1.13 | 0.11–12.1 |  |
| Any remission | 0.61 | 0.12–3.18 |  |
| ***hypertensive disorder of pregnancy*** |  |  |  |
| No remission | Ref | Ref |  |
| Clinical remission on CS | 0.46 | 0.124–1.73 |  |
| Clinical remission off CS | NA | NA |  |
| Complete remission | 0.95 | 0.167–5.42 |  |
| Any remission | 0.49 | 0.15–1.58 |  |
| ***HELLP syndrome*** |  |  |  |
| No remission | Ref | Ref |  |
| Clinical remission on CS | 0.90 | 0.05–12.9 |  |
| Clinical remission off CS | NA | NA |  |
| Complete remission | NA | NA |  |
| Any remission | 0.62 | 0.04–10.2 |  |
| ***Oligohydramnios*** |  |  |  |
| No remission | Ref | Ref |  |
| Clinical remission on CS | 0.13 | 0.01–1.13 |  |
| Clinical remission off CS | 1.03 | 0.10–10.5 |  |
| Complete remission | NA | NA |  |
| Any remission | 0.19 | 0.04–0.97 |  |
| **live birth** |  |  |  |
| No remission | Ref | Ref |  |
| Clinical remission on CS | 3.49 | 0.85–14.3 |  |
| Clinical remission off CS | NA | NA |  |
| Complete remission | 1.24 | 0.22–6.94 |  |
| Any remission | 2.98 | 0.89–9.94 |  |
| **preterm birth** |  |  |  |
| No remission | Ref | Ref |  |
| Clinical remission on CS | 0.84 | 0.24–2.92 |  |
| Clinical remission off CS | 0.75 | 0.076–7.44 |  |
| Complete remission | 0.56 | 0.06–5.39 |  |
| Any remission | 0.78 | 0.25–2.5 |  |
| **spontaneous abortion** |  |  |  |
| No remission | Ref | Ref |  |
| Clinical remission on CS | 1.84 | 0.16–21.2 |  |
| Clinical remission off CS | NA | NA |  |
| Complete remission | NA | NA |  |
| Any remission | 1.25 | 0.11–14.3 |  |
| **missed abortion** |  |  |  |
| No remission | Ref | Ref |  |
| Clinical remission on CS | NA | NA |  |
| Clinical remission off CS | NA | NA |  |
| Complete remission | 1.13 | 0.11–12.1 |  |
| Any remission | 0.20 | 0.02–2.02 |  |
| **Iatrogenic abortion** |  |  |  |
| No remission | Ref | Ref |  |
| Clinical remission on CS | 0.16 | 0.02–1.44 |  |
| Clinical remission off CS | NA | NA |  |
| Complete remission | 0.64 | 0.07–6.14 |  |
| Any remission | 0.23 | 0.04–1.22 |  |
| **Low birth weight** |  |  |  |
| No remission | Ref | Ref |  |
| Clinical remission on CS | 0.38 | 0.14–1.05 |  |
| Clinical remission off CS | 0.37 | 0.062–2.25 |  |
| Complete remission | 0.27 | 0.05–1.51 |  |
| Any remission | 0.36 | 0.14–0.92 |  |
| **SGA (%)** |  |  |  |
| No remission | Ref | Ref |  |
| Clinical remission on CS | 0.87 | 0.26–2.92 |  |
| Clinical remission off CS | 0.64 | 0.064–6.37 |  |
| Complete remission | NA | NA |  |
| Any remission | 0.67 | 0.21–2.15 |  |
| **Apgar.score.1m>7 (%)** |  |  |  |
| No remission | Ref | Ref |  |
| Clinical remission on CS | 2.74 | 0.24–31.8 |  |
| Clinical remission off CS | NA | NA |  |
| Complete remission | NA | NA |  |
| Any remission | 3.93 | 0.34–45.3 |  |
| **Apgar.score.5m>7 (%)** |  |  |  |
| No remission | Ref | Ref |  |
| Clinical remission on CS | NA | NA |  |
| Clinical remission off CS | NA | NA |  |
| Complete remission | NA | NA |  |
| Any remission | NA | NA |  |
| **Major malformation (%)** |  |  |  |
| No remission | Ref | Ref |  |
| Clinical remission on CS | 0.78 | 0.05–12.6 |  |
| Clinical remission off CS | NA | NA |  |
| Complete remission | NA | NA |  |
| Any remission | 0.53 | 0.32–8.80 |  |

APO, adverse pregnancy outcome; CS, corticosteroid; NA, not applicable; OR, odds ratio; SGA, small for gestational age

**Supplementary Table S7. Baseline characteristics in pregnant in remission at conception according to the serological activity**

| Factor | clinical remission with active serology | Clinical and serological remission | p-value |
| --- | --- | --- | --- |
| n | 35 | 24 |  |
| ***Epidemiological findings*** |  |  |  |
| Age at conception (years old) | 33.00 [29.00, 35.50] | 32.50 [28.75, 35.25] | 0.91 |
| Height (cm) | 156.00 [153.00, 163.00] | 159.50 [153.00, 161.75] | 0.73 |
| Weight (kg) | 48.00 [43.45, 54.40] | 48.95 [47.00, 55.85] | 0.31 |
| BMI | 20.00 [17.86, 21.12] | 19.89 [18.40, 21.35] | 0.48 |
| **Duration of SLE (days)** | **2849 [2146, 4230]** | **1760 [1152, 3296]** | **0.047** |
| ethnicity (%) | 32 (91.4) | 21 (87.5) | 0.68 |
| Smoking history (%) | 1 (2.9) | 3 (12.5) | 0.29 |
| **History of spontaneous abortion (%)** | **4 (11.4)** | **8 (33.3)** | **0.050** |
| History of use of hypertensive medication (%) | 0 (0.0) | 2 (8.3) | 0.16 |
| Multiparous (%) | 9 (26.5) | 12 (50.0) | 0.10 |
| Infertility treatment (%) | 11 (31.4) | 6 (25.0) | 0.77 |
| Planned pregnancy (%) | 35 (100) | 23 (95.8) | 0.41 |
| Thrombocytopenia at conception (%) | 0 (0.0) | 0 (0.0) | 1.0 |
| ***Organ manifestation*** |  |  |  |
| Joint and muscular manifestation (%) | 26 (74.3) | 12 (50.0) | 0.10 |
| Skin and mucocutaneous manifestation (%) | 29 (82.9) | 15 (62.5) | 0.13 |
| Renal manifestation (%) | 9 (25.7) | 3 (12.5) | 0.33 |
| Lupus nephritis class III/IV (%) | 4 (11.4) | 0 (0.0) | 0.14 |
| serositis (%) | 10 (28.6) | 5 (20.8) | 0.56 |
| neurological (%) | 6 (17.1) | 1 (4.2) | 0.22 |
| hematological (%) | 30 (85.7) | 23 (95.8) | 0.39 |
| ***Immunological findings*** |  |  |  |
| **Anti-dsDNA antibody (%)** | **33 (94.3)** | **11 (45.8)** | **<0.01** |
| Anti-RNP antibody (%) | 11 (39.3) | 7 (33.3) | 0.77 |
| Anti-Sm antibody (%) | 17 (50.0) | 6 (26.1) | 0.10 |
| Anti-Ro/SSA antibody (%) | 26 (76.5) | 14 (58.3) | 0.16 |
| Anti-La/SSB antibody (%) | 8 (27.6) | 2 (11.1) | 0.28 |
| LAC (%) | 5 (15.2) | 3 (12.5) | 1.0 |
| Anti-CL antibody (%) | 7 (20.0) | 10 (43.5) | 0.078 |
| Anti-Clβ2GPI antibody (%) | 5 (14.7) | 0 (0.0) | 0.070 |
| **Low C3 (%)** | **28 (80.0)** | **12 (50.0)** | **0.023** |
| Low C4 (%) | 30 (85.7) | 17 (70.8) | 0.20 |

Ab, antibody; BMI, body mass index; CL, cardiolipin; LAC, lupus anticoagulant; SLE, systemic lupus erythematosus

**Supplementary Table S8. Treatment regimen of pregnant in remission at conception according to the serological activity**

| Factor | Clinical remission with active serology | Clinical and serological remission | p-value |
| --- | --- | --- | --- |
| n | 35 | 24 |  |
| **GC (mg/day)** | **4.50 [2.50, 5.00]** | **0.50 [0.00, 5.00]** | **0.018** |
| HCQ (%) | 21 (60.0) | 11 (45.8) | 0.30 |
| Tac (%) | 10 (28.6) | 3 (12.5) | 0.21 |
| CyA (%) | 2 (5.7) | 0 (0.0) | 0.51 |
| AZA (%) | 4 (11.4) | 0 (0.0) | 0.14 |
| MMF (%) | 0 (0.0) | 0 (0.0) | 1.0 |
| MZR (%) | 0 (0.0) | 1 (4.2) | 0.41 |
| MTX (%) | 0 (0.0) | 0 (0.0) | NA |
| BEL (%) | 1 (2.9) | 0 (0.0) | 1.0 |
| RTX/CY/PE/IVIg (%) | 0 (0.0) | 0 (0.0) | NA |
| aspirin (%) | 16 (45.7) | 14 (58.3) | 0.43 |

AZA, azathioprine; BEL, belimumab; CY, cyclophosphamide; CyA, cyclosporine; GC, glucocorticoid; HCQ, hydroxychloroquine; IVIg, intravenous immunoglobulin; MMF, mycophenolate mofetil; MTX, methotrexate; MZR, MZR; NA, not available; PE, plasma exchange; PSL, prednisolone; RTX, rituximab; Tac, tacrolimus

**Supplementary Table S9. Baseline characteristics according to the glucocorticoid dosage at conception**

|  | Glucocorticoid dosage at conception | | |
| --- | --- | --- | --- |
| Factor | PSL< 7.5mg | PSL≥ 7.5mg | p value |
| n | 64 | 32 |  |
| ***Epidemiological findings*** |  |  |  |
| Age at conception | 33.0 [29.0, 36.0] | 33.5 [30.8, 36.0] | 0.52 |
| BMI | 19.9 [18.2, 21.4] | 20.0 [19.1, 21.2] | 0.56 |
| Duration of SLE | 2491.0 [1431.8, 4240.8] | 2741.50 [1887.5, 5219.3] | 0.64 |
| Smoking history | 5 (7.8) | 2 (6.2) | 1.0 |
| previous spontaneous abortion | 13 (20.3) | 5 (15.6) | 0.78 |
| previous Anti-hypertensive med use | 4 (6.2) | 2 (6.2) | 1.0 |
| Multiparous | 24 (8.1) | 12 (37.5) | 1.0 |
| Infertility treatment | 20 (31.2) | 7 (21.9) | 0.47 |
| Any flare at conception | **0 (0.0)** | **6 (18.8)** | **<0.01** |
| Doria remission at conception | **59 (92.2)** | **0 (0.0)** | **<0.01** |
| ***Organ manifestation*** |  |  |  |
| Joint/muscular manifestation | 42 (65.6) | 22 (68.8) | 0.82 |
| Skin/mucocutaneous manifestation | 48 (75.0) | 23 (71.9) | 0.81 |
| Renal manifestation | 17 (26.6) | 8 (25.0) | 1.0 |
| lupus nephritis class III/IV | 6 (9.4) | 3 (9.4) | 1.0 |
| Serositis | 16 (25.0) | 6 (18.8) | 0.61 |
| neurological manifestation | **9 (14.1)** | **0 (0.0)** | **0.03** |
| hematological manifestation | 56 (87.5) | 25 (78.1) | 0.25 |
| ***Immunological profile*** |  |  |  |
| Anti-dsDNA Ab | **49 (76.6)** | **14 (43.8)** | **<0.01** |
| Anti-RNP Ab | 19 (35.2) | 11 (55.0) | 0.18 |
| Anti-Sm Ab | 25 (40.3) | 6 (23.1) | 0.15 |
| Anti-SSA Ab | 43 (68.3) | 18 (56.2) | 0.27 |
| Anti-SSB Ab | 10 (19.6) | 2 (9.1) | 0.33 |
| LAC | 9 (14.5) | 3 (9.7) | 0.75 |
| Anti-CL Ab | **19 (30.6)** | **3 (10.3)** | **0.04** |
| Anti-CLβ2GPI Ab | 5 (7.9) | 3 (9.7) | 1.0 |
| low C3 | 44 (68.8) | 19 (63.3) | 0.64 |
| low C4 | 51 (79.7) | 26 (86.7) | 0.57 |

Ab, antibody; BMI, body mass index; CL, cardiolipin; LAC, lupus anticoagulant; SLE, systemic lupus erythematosus **Supplementary Table S10. Treatment regimen at conception according to the glucocorticoid dosage at conception**

|  | Glucocorticoid dosage at conception | | |
| --- | --- | --- | --- |
| Factor | PSL< 7.5 mg | PSL≥ 7.5 mg | p-value |
| n | 64 | 32 |  |
| **GC (mg/day)** | **4.00 [0.00, 5.00]** | **10.00 [9.88, 15.00]** | **<0.01** |
| **HCQ (%)** | **35 (54.7)** | **11 (34.4)** | **0.08** |
| Tac (%) | 16 (25.0) | 10 (31.2) | 0.63 |
| CyA (%) | 2 (3.1) | 0 (0.0) | 0.55 |
| AZA (%) | 4 (6.2) | 2 (6.2) | 1.0 |
| MMF (%) | 0 (0.0) | 1 (3.1) | 0.33 |
| MZR (%) | 1 (1.6) | 0 (0.0) | 1.0 |
| BEL (%) | 1 (1.6) | 2 (6.2) | 0.26 |
| MTX/RTX/CY/PE/IVIg (%) | 0 (0.0) | 0 (0.0) | NA |
| **aspirin (%)** | **34 (53.1)** | **8 (25.0)** | **0.010** |

AZA, azathioprine; BEL, belimumab; CY, cyclophosphamide; CyA, cyclosporine; GC, glucocorticoid; HCQ, hydroxychloroquine; IVIg, intravenous immunoglobulin; MMF, mycophenolate mofetil; MTX, methotrexate; MZR, MZR; NA, not available; PE, plasma exchange; PSL, prednisolone; RTX, rituximab; Tac, tacrolimus

**Supplementary Table S11. multivariate analysis on risk of averse pregnancy outcome according to the** **glucocorticoid dosage (prednisolone equivalent) ≥7.5 mg/day at conception**

|  | Multivariate logistic regression model  analysis 1 | | | Multivariate logistic regression model  analysis 2 | | | Multivariate logistic regression model  analysis 3 | | | Multivariate logistic regression model  analysis 4 | | | Multivariate logistic regression model  analysis 5 | | |
| --- | --- | --- | --- | --- | --- | --- | --- | --- | --- | --- | --- | --- | --- | --- | --- |
| Factor | aOR^a^ | 95% CI | p value | aOR^a^ | 95% CI | p value | aOR^a^ | 95% CI | p value | aOR^a^ | 95% CI | p value | aOR^a^ | 95% CI | p value |
| **Overall APO (%)** | **3.11** | **1.20–8.04** | **0.019** | **2.63** | **1.0-6.89** | **0.049** | **2.76** | **1.05-7.25** | **0.039** | 1.49 | 0.51-4.36 | 0.47 | 1.26 | 0.42-3.78 | 0.68 |
| **Maternal APO (%)** | 2.78 | 0.98–7.88 | 0.055 | 2.43 | 0.83-7.14 | 0.11 | 2.20 | 0.74-6.50 | 0.16 | 1.01 | 0.28-3.73 | 0.98 | 0.76 | 0.20-2.91 | 0.69 |
| **Neonatal APO (%)** | **2.91** | **1.14–7.38** | **0.025** | **2.84** | **1.06-7.59** | **0.037** | **3.08** | **1.18-8.06** | **0.02** | 1.92 | 0.68-5.44 | 0.22 | 1.86 | 0.62-5.52 | 0.27 |
| PROMISSE APO (%) | 1.59 | 0.56–4.50 | 0.38 | 1.51 | 0.53-4.32 | 0.45 | 1.80 | 0.62-5.2 | 0.28 | 2.07 | 0.64-6.74 | 0.23 | 2.15 | 0.65-7.14 | 0.21 |

a adjusted odds ratio of glucocorticoid dosage (prednisolone equivalent) ≥7.5 mg/day for each APO

analysis 1: adjusted for renal manifestation, hydroxychloroquine prescription, and aspirin prescription at conception.

analysis 2: adjusted for renal manifestation, hydroxychloroquine prescription, aspirin prescription at conception, and LAC positivity

analysis 3: adjusted for renal manifestation, hydroxychloroquine prescription, aspirin prescription at conception, and thrombocytopenia at conception

analysis 4^※^: adjusted for renal manifestation, hydroxychloroquine prescription, aspirin prescription at conception, and pregnancy planning

analysis 5^※^: adjusted for renal manifestation, hydroxychloroquine prescription, aspirin prescription at conception, and LAC positivity, thrombocytopenia at conception and pregnancy planning

aOR; adjusted odds ratio APO; adverse pregnancy outcome, CI; confidence interval, PROMISSE; Predictors of Pregnancy Outcome: Biomarkers in Antiphospholipid Antibody Syndrome and Systemic Lupus Erythematosus, SGA; small for gestational age

※SLE disease stability is one of the factors to allow patients to plan pregnancy. Given this context, we consider 'planned pregnancy' as a potential mediator in the relationship between remission and APOs, rather than a confounder.
